# Management of female para-urethral cyst with dyspareunia: a case report

**DOI:** 10.1186/s13256-024-04984-4

**Published:** 2025-01-13

**Authors:** Manoj Kumar Deepak, R. M. Meyyappan, T. Senthil Kumar, J. Saravanan

**Affiliations:** Department of Urology, SRM Institute of Science and Technology, SRM Nagar, Chengalpattu, Kattankulathur, Tamilnadu 603203 India

**Keywords:** Dyspareunia, Para urethral cysts, Skene gland cyst

## Abstract

**Background:**

The diagnosis and management of female genital conditions (Rodriguez et al. in Clin Anat 34(1):103–107, 2020. 10.1002/ca.23654) are often challenging. The atypical presentations, combined with patient hesitancy to be subjected to an examination by a male urologist, are factors that limit a timely diagnosis. Para-urethral cysts (Pastor and Chmel in Int Urogynecol J 29(5):621–629, 2018. 10.1007/s00192-017-3527-9) are often incidentally detected by gynecologists during pelvic examination for other reasons. Patients rarely present with complaints of lower urinary tract symptoms and dyspareunia affecting sexual life. Diagnosis in most instances can be made by physical examination but often a detailed evaluation with ultrasonography, voiding cystourethrogram, computed tomography, or magnetic resonance imaging is needed. The definitive management of symptomatic para-urethral cysts is through surgical excision.

**Objective:**

This report aims to reflect clinically upon a rare pathology of the female genital system.

**Case presentation:**

We present the case of a 36-year-old, sexually active, Indian (Asian) woman with a 6-month history of progressively worsening lower urinary tract symptoms, consisting of dysuria, post-micturition dribble, increased urination frequency, and significant dyspareunia. Physical examination in the lithotomy position revealed a cystic lesion located in the midline slightly to the left of the anterior vaginal wall. Magnetic resonance imaging also revealed a T2/T1 hyperintense lesion located below the level of the pubic symphysis. The patient was posted for exploration under anesthesia and the cyst was excised completely. The histopathology findings were consistent with para-urethral gland cyst with ulceration and squamous metaplasia.

**Conclusion:**

Any lower urinary tract symptoms in a woman needs thorough clinical examination. Association of para-urethral cyst with lower urinary tract symptoms and dyspareunia is rare, and if present, always warrants surgical excision.

## Background and introduction

The diagnosis and management of female genital conditions [1] from a urologist’s standpoint are often challenging Atypical presentations in terms of symptoms combined with patient hesitancy to be subjected to an examination by a male urologist, are factors that limit a timely diagnosis [2]. Para-urethral cysts [3] are often incidentally detected by gynecologists during pelvic examination for other reasons. Patients rarely present with complaints of lower urinary tract symptoms (LUTS) and dyspareunia [4]. Diagnosis [5, 6] in most instances can be made by physical examination but often a detailed evaluation with ultrasonography (US), voiding cystourethrogram (VCUG), computed tomography (CT), or magnetic resonance imaging (MRI) is needed to rule out other possible causes [5]. Management of symptomatic para-urethral cysts is by surgical excision.

### Aim

This report aims to reflect clinically upon a rare pathology of the female genital system.

### Case presentation

We present the case of a 36-year-old, sexually active, Indian (Asian) woman with a 6-month history of progressively worsening lower urinary tract symptoms, including dysuria, post-micturition dribble, increased frequency of urination, and significant dyspareunia. Over the past few months, she had consulted several physicians, who had treated her with antibiotics and analgesics. When she presented to our clinic, a detailed history revealed that she had experienced a sensation of a protruding mass in the vaginal area over the past few years, with dyspareunia, along with lower urinary tract symptoms (LUTS). On physical examination in the lithotomy position, we observed a cystic lesion located midline and slightly to the left of the anterior vaginal wall, displacing the urethral orifice anteriorly and giving it a *slit-like* appearance.

### Investigations

1. Vaginal ultrasound revealed a solitary 4 cm cyst localized in the distal urethra.
2. Pelvic MRI also revealed a T2/T1 hyperintense lesion located below the level of the pubic symphysis.

The patient was posted for exploration under anesthesia.

**Fig. 1:**
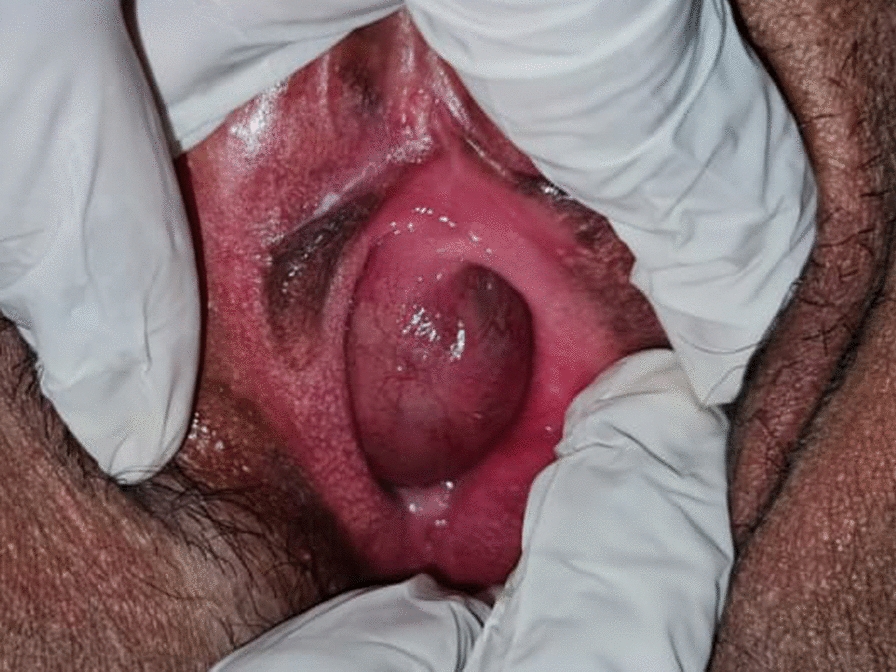
Clinical examination picture showing a cystic swelling pushing the urethral meatus

**Fig. 2:**
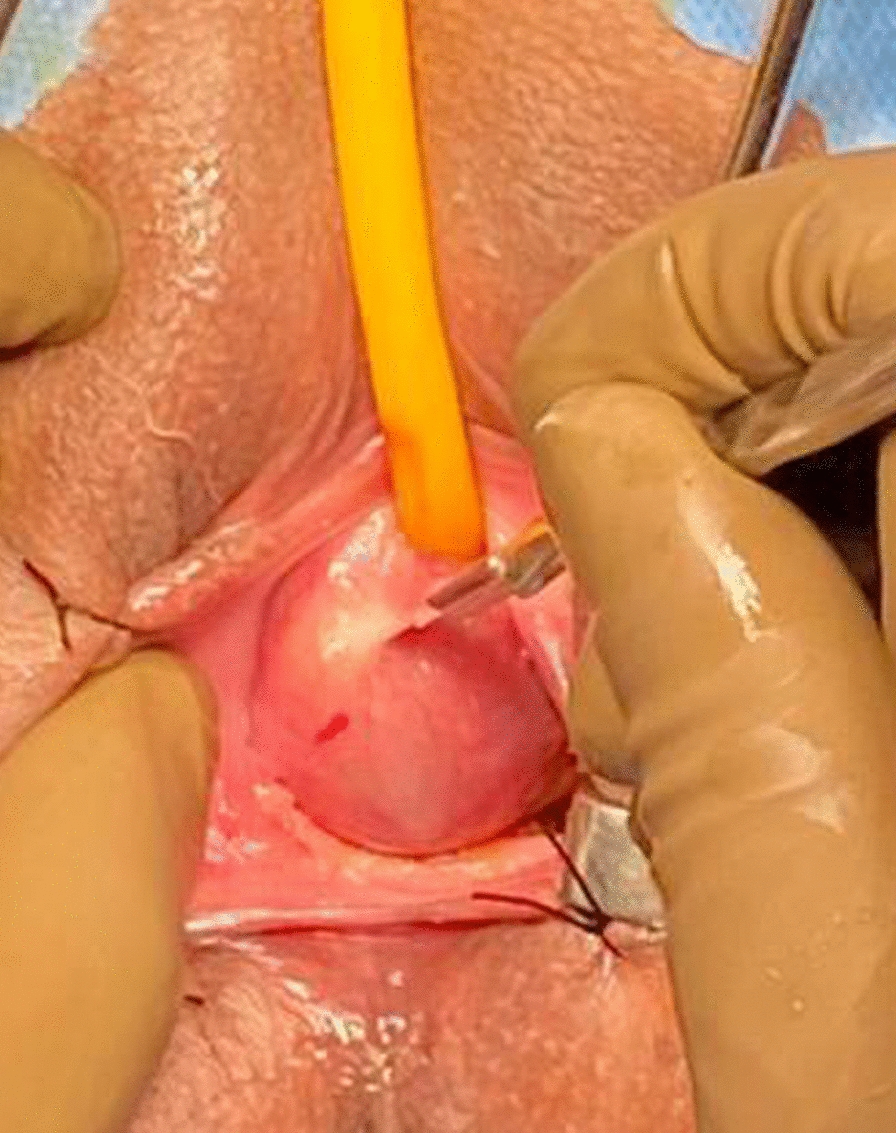
Incision marking around the cyst

**Fig. 3:**
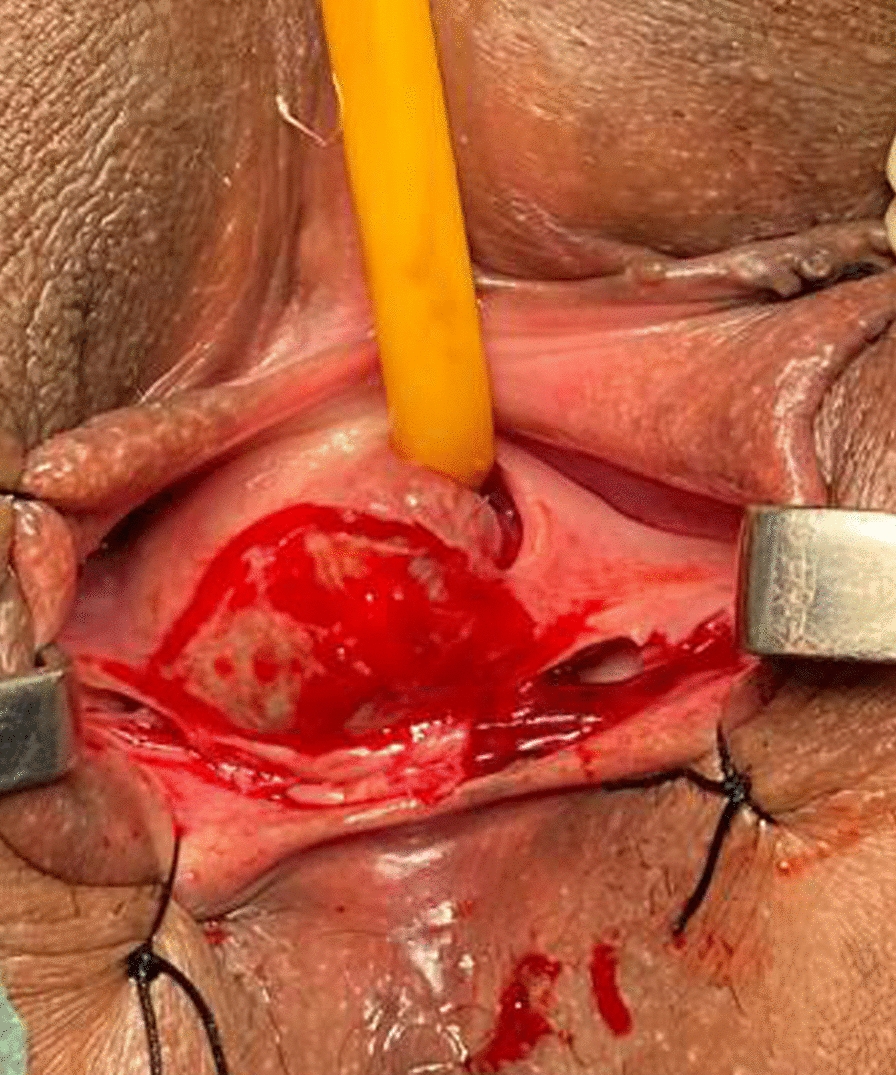
Flaps raised and cyst excised

The procedure was done under spinal anesthesia, an 18 Fr Foley catheter was inserted into the bladder following sterile preparation and draping. A speculum examination was performed, and the labia were retracted using stay sutures (Fig. 1). The incision site was carefully marked on the vaginal wall (Fig. 2). Vaginal flaps were raised, and the cyst was separated from surrounding tissues using sharp dissection (Fig. 3).

**Fig. 4:**
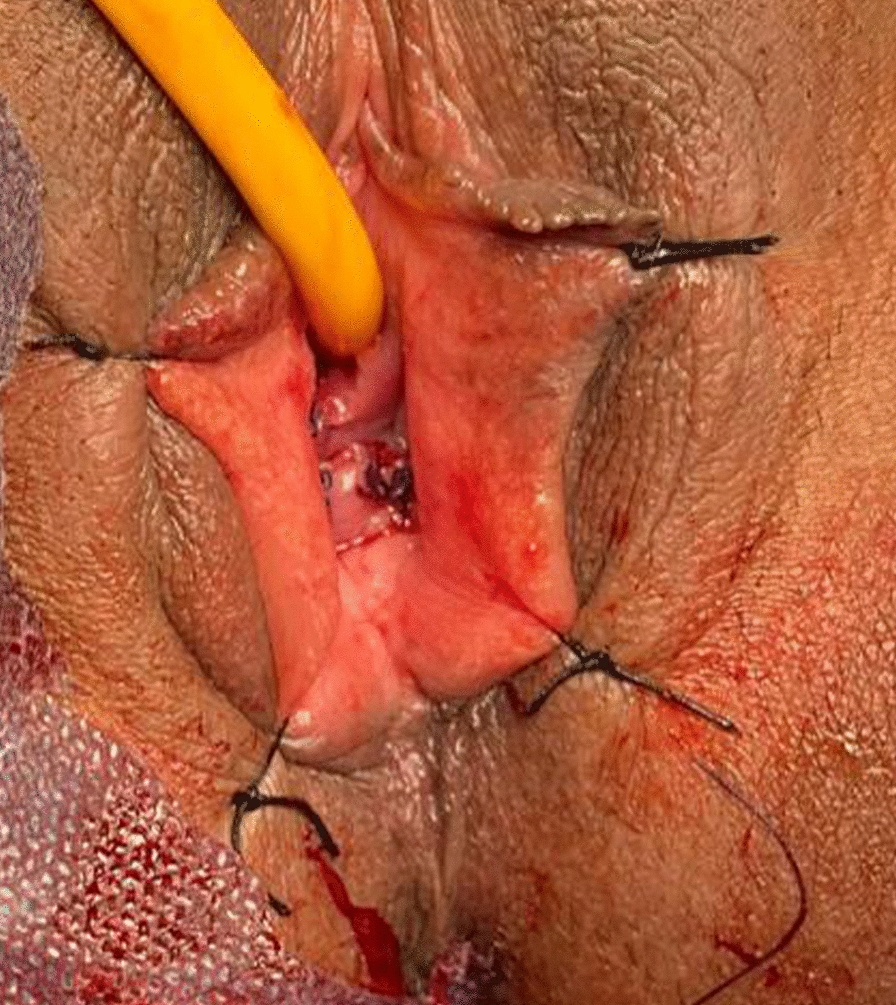
Postoperative image showing complete excision

Upon opening the cyst, thick contents were identified and completely drained. A thorough lavage was performed using povidone-iodine, followed by the complete excision of the cyst wall (Fig. 4). The excised specimen was sent for histopathological evaluation.

The patient was discharged in good condition with instructions to maintain local hygiene and abstain from sexual intercourse during the healing period. Histopathological analysis revealed findings consistent with a **para-urethral gland (Skene’s gland) cyst** exhibiting ulceration and squamous metaplasia.

During monthly follow-ups over a 6-month period, the patient demonstrated significant improvement in voiding patterns and reported no dyspareunia.

## Discussion

The **Skene glands** [5], also known as **para-urethral or lesser vestibular glands**, are located around the lower end of the urethra. These glands were first described in 1672 by Regnier de Graaf and later by the French surgeon Alphonse Guérin (1816–1895). They were named after the Scottish gynecologist *Alexander Skene*, who detailed their anatomy and function in Western medical literature in 1880.

The two Skene gland ducts open into the vulvar vestibule on either side of the urethral opening, secreting a milk-like fluid [7, 8] that is an ultrafiltrate of plasma. This secretion, released during vaginal stimulation, contains prostate-specific antigen (PSA) [7–9], acid phosphatase, and high concentrations of glucose and fructose. Embryologically, the Skene glands are homologous to the male prostate, sharing a common developmental origin.

Disorders of the Skene glands include:

- Infection: known as skenitis, urethral syndrome, or female prostatitis.
- Skene’s duct cyst [10]: typically secondary to obstruction of the ducts and lined by stratified squamous epithelium.
- Trichomoniasis: the Skene glands can act as a reservoir for pathogens, such as *Trichomonas vaginalis*.

The differential diagnosis of a Skene gland cyst includes ectopic ureterocele, pelvic organ prolapse, and urethral diverticulum. **Surgical excision** remains the most effective treatment for para-urethral cysts, offering consistent results. Alternative methods, such as waiting for spontaneous rupture, needle aspiration, or marsupialization, are less commonly employed and lack definitive outcomes.

## Limitations

This case report describes the presentation and management of a single case, reflecting the experience of our institute. Broader studies with larger sample sizes are needed to validate the findings and strengthen the evidence base for the diagnosis and management of Skene gland disorders.

## Conclusion

Any LUTS in a woman needs a thorough clinical examination. Better doctor–patient communication, trust building, and always utilizing a chaperone during clinical examination are key factors that help prevent patient hesitancy and provide the right diagnosis.

Para-urethral cysts as a cause of LUTS and dyspareunia are rarely reported in literature. These cysts are usually asymptomatic or may develop infection and abscess formation. Association of para-urethral cyst with LUTS and dyspareunia is rare and always warrants surgical excision if present.

## Data Availability

Available.
